# P-394. Prevention Of Infections In Cardiac Surgery (PICS)-Prevena Study – a pilot/vanguard factorial cluster cross-over RCT

**DOI:** 10.1093/ofid/ofae631.595

**Published:** 2025-01-29

**Authors:** Thomas C C Scheier, Richard Whitlock, Mark Loeb, P J Devereaux, Shun Fu Lee, Dominik Mertz

**Affiliations:** Population Health Research Institute, Hamilton, Ontario, Canada; McMaster university, Hamilton, Ontario, Canada; McMaster University, Hamilton, ON, Hamilton, Ontario, Canada; McMaster University, Hamilton, Ontario, Canada; McMaster University, Hamilton, Ontario, Canada; McMaster University, Hamilton, Ontario, Canada, Hamilton, ON, Canada

## Abstract

**Background:**

Sternal surgical site infections (s-SSI) after cardiac surgery can lead to significant morbidity, mortality, and costs. Among cardiac surgery patients, the effects of negative pressure wound management is unknown.

**Table 1:**
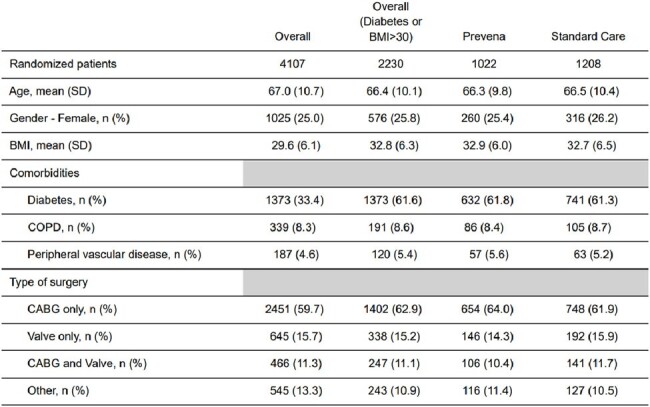
Baseline Characteristics BMI= Body Mass Index; COPD= Chronic obstructive pulmonary disease; CABG= Coronary artery bypass graft; SD= Standard deviation

**Methods:**

The PICS-PREVENA Vanguard study, a 2x2 factorial, open label, cluster-randomized crossover trial, was conducted at 2 hospitals in Ontario, Canada (NCT03402945). We randomized each site to one of four sequences of the four study arms – each arm combining either cefazolin mono vs. combination prophylaxis with vancomycin (not analyzed) with either standard wound dressing vs. the 3M Prevena incision management system (Prevena). Only diabetic or obese patients (BMI >30kg/m^2^) were eligible for the latter comparison. For this population, we report the feasibility and efficacy endpoints of the Prevena versus standard wound dressing interventions. Feasability was assessed by adherence to the study protocol and loss to follow-up. The primary efficacy outcome was a composite of deep and organ-space s-SSI within 90-days after surgery. We used mixed logistic regression, accounting for clusters as a random effect for analysis. Funding: KCI USA, Inc.

**Table 2:**
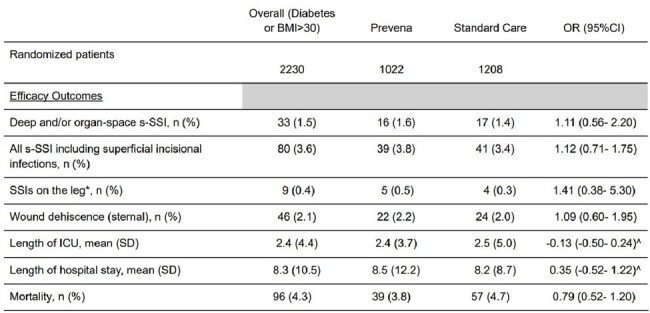
Outcomes at end of follow up (90 days) BMI= Body Mass Index; s-SSI= Sternal surgical site infections; SSI= surgical site infections; ICU= intensive care unit; OR= odds ratio; SD= Standard deviation; CI= confidence interval ^ Mean Difference * For patients with open venous saphenous harvest

**Results:**

Among the 4107 included patients, 2230 were obese or diabetic. Of these patients, 1208 underwent surgery during a standard wound dressing period, and 1022 during a Prevena allocated period (Figure and Table 1). Of the latter, 696 (68.1%) had Prevena applied. Loss to follow up was 3.6% for obese or diabetic patients.

Deep and organ-space s-SSI occurred in 16 (1.6%) and 17 (1.4%) in the Prevena and standard wound dressing allocated periods, respectively (OR= 1.11, 95% CI: 0.56- 2.20). Other clinical outcomes (Table 2) did not suggest a difference: all s-SSI (3.8% vs. 3.4%, OR: 1.12, 95% CI: 0.71- 1.75), SSI on legs (0.5% vs. 0.3%, OR: 1.41, 95% CI: 0.38- 5.30), and 90-day mortality (3.8% vs. 4.7%; OR: 0.79 (95% CI: 0.52- 1.20). An on-treatment analysis showed similar results.

**Figure 1:**
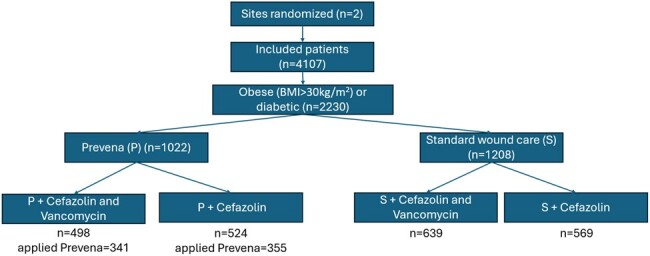
Treatment assignment BMI= Body Mass Index

**Conclusion:**

The vanguard study showed challenges with introducing a novel technology as standard of care with only a 68% compliance overall, with non-compliance mostly driven by one of the sites. No conclusions should be drawn regarding the efficacy of Prevena, as this Vanguard phase was not powered for these outcomes.

**Disclosures:**

**Richard Whitlock, PhD**, Abbott: Grant/Research Support|Atricure: Grant/Research Support|CytoSorbents: Grant/Research Support **PJ Devereaux, MD, PhD**, Abbott Diagnostics: Advisor/Consultant|Abbott Diagnostics: Grant/Research Support|AOP Pharma: Grant/Research Support|Astra Zeneca: Advisor/Consultant|Bayer: Advisor/Consultant|CloudDX: Monitoring Devices|Quidel Canada: Advisor/Consultant|Renibus: Advisor/Consultant|Roche Canada: Advisor/Consultant|Roche Diagnostics: Grant/Research Support|Siemens: Grant/Research Support|Trimedic: Advisor/Consultant **Dominik Mertz, MD, MSc**, KCI Inc. USA: Grant/Research Support

